# Lipid-Based Drug Delivery Systems: Concepts and Recent Advances in Transdermal Applications

**DOI:** 10.3390/nano15171326

**Published:** 2025-08-28

**Authors:** Lefkothea Antonara, Efstathia Triantafyllopoulou, Maria Chountoulesi, Natassa Pippa, Paraskevas P. Dallas, Dimitrios M. Rekkas

**Affiliations:** Section of Pharmaceutical Technology, Department of Pharmacy, School of Health Sciences, National and Kapodistrian University of Athens, 15784 Athens, Greece; lefkanto@pharm.uoa.gr (L.A.); efstrian@pharm.uoa.gr (E.T.); mchountoules@pharm.uoa.gr (M.C.); dallas@pharm.uoa.gr (P.P.D.); rekkas@pharm.uoa.gr (D.M.R.)

**Keywords:** lipid nanoparticles, transdermal, drug delivery nanosystems, skin administration, liposomes, ethosomes, nanostructured lipid carriers, Quality by Design, solid lipid nanoparticles, transferosomes

## Abstract

Lipid-based nanocarriers are ideal drug delivery systems for transdermal administration due to their biocompatibility and biodegradability. Their lipophilicity and/or similarity to the natural lipids of the epidermis enable intermolecular interactions with the lipid membrane and therefore result in effective passage through the skin. The purpose of this review is to focus on lipid-based drug delivery nanoplatforms administered via the transdermal route by summarizing the most recent developments with the intention of fast clinical translation. Liposomes, solid lipid nanoparticles (SLNs), nanostructured lipid carriers (NLCs), ethosomes, and transfersomes exhibit ideal physicochemical characteristics and encapsulation efficiency to enhance the effectiveness of the incorporated Active Pharmaceutical Ingredients (APIs). The state of the art for fabricating transcutaneous lipid drug delivery nanoparticles and the strategies for overcoming the current obstacles, as well as the added value of novel formulations, will be discussed within the scope of Quality by Design applications. The limitations and challenges that still exist will also be considered.

## 1. Introduction

The transdermal route is favorable for drug administration, especially due to its non-invasive character. Other benefits include its controlled release capability, the avoidance of presystemic metabolism (first-pass effect), and the reduction in side effects [1,2]. However, it is not often selected due to the limitations that still exist. Among other functionalities, skin is a physiological barrier, being impervious to some compounds and thus limiting drug permeation, while it seems that there is also skin-mediated metabolism. Bearing in mind that skin is a dynamic system, the degree of a drug’s penetration is dependent on skin condition and hydration as well [1,2]. Additionally, Active Pharmaceutical Ingredients (APIs) that are ionizable molecules and/or have a molecular weight over 500 Dalton face difficulties permeating the skin [3]. Even in the case of APIs that could penetrate the stratum corneum successfully, passing through the skin multilayer can be a slow process, leading to ineffective therapeutic drug levels [2]. Strategies to exceed such barriers involve physical and chemical methods. Ionto-, sono-, thermo-, and magnetophoresis; electroporation; microneedles; and microdermabrasion are the existing physical methodologies, whereas chemical techniques include the usage of prodrugs or excipients that enhance skin permeation, such as terpenes, alcohols, glycols, etc. [4,5,6,7]. Both approaches show several disadvantages including irritation and other side effects [4]. Alternatively, the use of novel formulations utilizing nanoparticulate systems as drug delivery platforms is a promising strategy for transcutaneous administration.

Nano-drug delivery systems are innovative carriers comprising a broad spectrum of natural and/or chemical materials, such as inorganic, organic, or both. Their common feature—and main advantage—is their size, as being on the nanoscale results in them having an increased surface area [8]. They can be tailor-made according to the needs of their intended use and utilized for the encapsulation of either APIs with low molecular weight or macromolecules. In addition, they have the capability of being able to safeguard their load from degradation, promote their modified release, and improve their pharmacokinetics. Their flexibility permits the incorporation of both hydrophilic and hydrophobic APIs and the co-loading of more than one compound with no compatibility issues, and they have excellent surface functionalization and stimuli-responsive capability. Apart from drug delivery, they can be utilized for imaging and theragnostic purposes as well. Therefore, they are identified as a smart multifunctional drug delivery system, and they are selected to safely, effectively, and selectively deliver their cargo [8,9,10]. However, there are still important considerations, especially regarding their safety due to their nanoparticulate nature as well as difficulties in their clinical translation due to heterogeneity in populations and the clinical gap between studies of humans and animals [8,11]. Referring to transdermal administration, the different categories of nanoparticles that can be found in the literature are organic ones, including lipid and polymer-based nanosystems; inorganic ones, such as metallic (gold nanoparticles, etc.); and carbon nanostructures and nanogels (or nanoemulsions) [12,13]. Among the most popular nanovectors are lipid drug delivery systems due to their advantages that are going to be discussed in detail.

The purpose of this review is to focus on lipid-based drug delivery nanoplatforms administrated via the transdermal route by summarizing the most recent developments with the intention of fast clinical translation. More specifically, the systems will be analyzed in pharmaceutical technology terms by describing the different types of lipid-based drug delivery systems for transdermal administration, as well as the Quality by Design approach applied during the development of lipid-based transdermal products, for the rational design of their formulation and manufacturing process. The state of the art for fabricating transcutaneous lipid drug delivery nanoparticles and the strategies for overcoming the current obstacles, as well as the added value of novel formulations, will be discussed thoroughly. Last but not least, case studies found in the literature about pre- and clinical studies on these systems will be referenced, while the current status and some considerations of their regulatory framework will also be described. The limitations and challenges that still exist will also be considered. The research methodology in this review work included a search conducted in the electronic databases “PubMed” and “Google Scholar” for articles published within the last 10 years, from 2015 to 2025. The search terms were the English words “transdermal administration” AND “lipid-based nanoparticles”, along with specific keywords, such as “liposomes”, “ethosomes”, “SLNs”, “NLC”, and “transferosomes”.

## 2. Anatomy of the Skin: Challenges for Formulation Strategies

**Figure 1:**
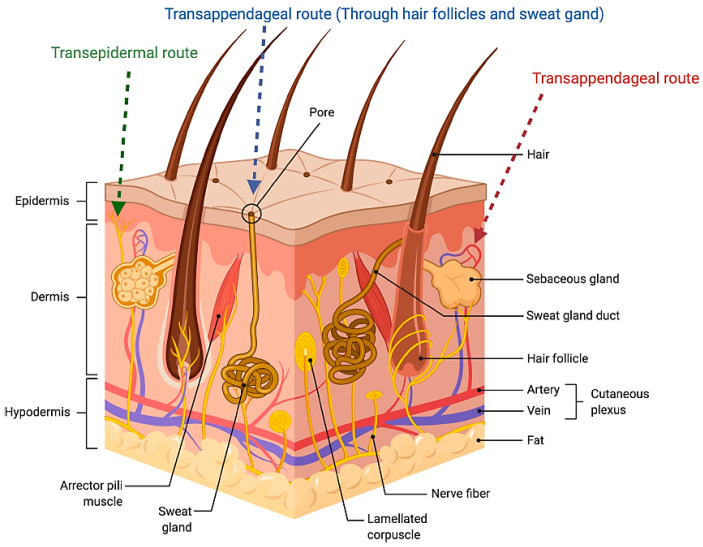
Schematic illustration of the skin layer showing penetration routes of the drug administered through the skin [15].

The skin represents the largest organ of the human body, accounting for approximately 10% of total body mass in an average individual. It acts as a protective barrier between the body and the outside world, shielding against harmful ultraviolet (UV) radiation, chemicals, allergens, and microorganisms, while also preventing the loss of water and essential nutrients. In addition to its protective function, the skin plays a key role in regulating body temperature and blood pressure, helping to maintain internal balance. It also serves as a sensory organ, detecting and responding to external stimuli such as touch, pressure, pain, and temperature [14] (Figure 1).

Human skin is composed of four primary layers, arranged from the outermost to the innermost layer, i.e., the stratum corneum, the viable epidermis, the dermis, and subcutaneous tissues. Embedded within these layers are various appendages such as hair follicles and sweat glands, which are visible on the skin’s surface. The stratum corneum consists of the outermost surface of the skin, with a thickness of 10–20 μm, functioning as the primary barrier of the skin. This layer is responsible for the regulation of the water loss and the prevention of the permeation of potentially harmful substances and microorganisms from the skin surface. Structurally, the stratum corneum is often compared to a brick wall, where the corneocytes function as the “bricks” and the surrounding lamellar lipids act as the “mortar” [14]. Corneocytes are flattened dead cells that have lost their nuclei as a result of keratinization, and they mainly consist of keratin (at a percentage of 70–80%) and a natural moisturizing factor. Their high water content allows them to swell, which helps maintain the elasticity of the stratum corneum and prevents the development of cracks in the skin [16].

The viable epidermis is a multilayered region whose thickness varies depending on the anatomical site. It consists of four different layers, i.e., stratum lucidum (encountered in areas with thick skin), stratum granulosum, stratum spinosum, and stratum basale. Keratinocytes are the main cell population, while melanocytes, Langerhans cells, and Merkel cells can be encountered at smaller percentages [16]. Melanocytes are responsible for the skin’s pigmentation by producing melanin, a high-molecular-weight polymer whose primary function is the protection of the skin from the effects of harmful UV radiation. Langerhans cells are a part of the immune system with the main function of protecting the skin from antigens by sensitizing T cells in the regional lymph nodes. Finally, Merkel cells are involved in sensation, as they are associated with nerve endings.

The dermis is a layer of 2–5 mm thickness connecting the epidermis and the subcutaneous tissue. Its extensive network of collagen fibrils provides support, while the dense elastic connective tissue provides elasticity and flexibility to the skin. Due to its hydrophilic structure, the dermis is not considered an effective barrier to the permeation of most drugs, apart from the ones that are highly lipophilic. In this region of the skin, several structures and appendages can be encountered, such as blood and lymph vessels, nerve endings, hair follicles, and sebaceous and sweat glands. Finally, the subcutaneous tissue is a lipid layer acting as a heat insulator as well as protecting against mechanical pressure. It also functions as energy storage, which can be available when necessary [14].

## 3. Lipid-Based Systems as a Strategy for Permeation Enhancement

As already stated in the previous section, the barrier function of the stratum corneum is crucial for the prevention of extensive water loss from the body and subsequently the protection of life. However, this exact barrier formation is also responsible for the limited drug permeation, which leads to difficulty in achieving sufficient therapeutic dosages systemically. As a result, various methods of permeation enhancement have been developed through the years and are categorized as passive and active [17]. While active techniques implement the application of a physical force for the breakage of the stratum corneum’s structure, passive ones are based on the utilization of chemical compounds such as ionic pairs, eutectic mixtures, chemical enhancers, and lipid-based systems, with the latter gaining an increasing interest in recent decades.

Lipids are a wide category favoring the formation of different types of drug delivery systems with versatile properties, amphiphilicity, and different mechanisms to permeate the skin. Moreover, they could protect their cargo from different environmental conditions ameliorating its stability [2,6]. Therefore, they can incorporate APIs with challenging properties and/or sensitive molecules with high molecular weight (such as nucleic acids and proteins) [12]. Optimizing their physicochemical features or modifying their surface are efficient strategies for passive or active targeting and thus increased potency in the intended area with minimum side effects [4]. In this manner, they could improve skin permeation and enhance the passive transdermal absorption of APIs with non-ideal properties for this route of administration, such as antibiotics, etc. [1,18]. They could be suitable candidates for novel therapies as well. For example, they might be selected to deliver adjuvants for transdermal vaccination or to ameliorate the transcutaneous delivery of macromolecules [7,19].

Lipid-based drug delivery systems are nanoparticle-sized experimental models of biomembranes, which are extensively used as vehicles for the controlled, targeted, or non-targeted delivery of active compounds via topical or systemic routes [20]. They exhibit a series of advantages such as biocompatibility, improved physicochemical stabilization, and non-toxicity with the ability to incorporate both hydrophilic and hydrophobic active substances [21]. Although they have been extensively utilized as a means of drug permeation, their mechanism of action is not yet fully clarified. Research studies indicate four potential mechanisms of permeation enhancement, i.e., (I) intact transport of the loaded molecules through the various layers of the skin, (II) functioning as permeation enhancers by liquefaction of the stratum corneum membrane, (III) exchange of the active substance during the fusion of the lipid bilayer with the surface of the skin’s cells, and/or (IV) absorption form the skin appendages such as hair follicles and sweat glands. In general, the mechanism(s) taking place are mostly dependent on the composition and the size of the particles. Specifically, particles of sizes less than 300 nm achieve a release of their content to some degree at the deepest skin layers, with maximum values achieved at particle sizes of less than or equal to 70 nm [22].

A comparative analysis of the lipid-based drug delivery systems highlights significant differences in their skin permeation mechanisms. Specifically, liposomes, composed mainly of phospholipids, remain located at the upper skin layers with little permeation into the deepest layers. Imaging studies such as confocal laser scanning microscopy (CLSM) [23,24] and in vitro tape stripping confirm their restricted diffusion profile [25]. On the contrary, transfersomes, which include edge activators to enhance vesicle deformability, can penetrate more effectively through the intercellular lipid matrix of the stratum corneum by changing their shape to pass through the narrow channels. This mechanism is supported by ex vivo skin permeation studies and CLSM [26,27]. However, their penetration depth is yet more limited in comparison to ethosomes, probably due to their larger size.

Ethosomes, another advanced vesicular system containing high concentrations of ethanol, facilitate deep skin penetration by fluidizing the SC lipids and enhancing the flexibility of the vesicle membrane. CLSM, transmission electron microscope (TEM), and Raman spectroscopy have provided strong visual evidence of their enhanced skin permeation compared to conventional forms [28,29].

Lipid-drug delivery systems such as solid lipid nanoparticles (SLNs) and nanostructured lipid carriers (NLCs) offer alternative strategies for dermal delivery. SLNs, consisting entirely of solid lipids, form an occlusive layer on the skin surface that enhances hydration and provides a slow-release profile, while NLCs—comprising a mixture of solid and liquid lipids—create a less-ordered lipid matrix that allows for improved drug loading and a more flexible structure. These findings are supported by ex vivo permeation models [30,31] and CLSM [32] imaging results. Overall, in the case where penetration at the deeper skin layers is desired, transfersomes, ethosomes, and NLCs constitute the most promising choices.

## 4. Types of Lipid-Based Drug Delivery Systems for Transdermal Administration

As already stated, the lipid-based systems can be divided into a number of categories based on their composition. Specifically, liposomes, solid lipid nanoparticles (SLNs), nanostructured lipid carriers (NLCs), ethosomes, transfersomes, niosomes, cubosomes, archaesomes, invasomes, penetration enhancer-containing vesicles (PEVs), and several other types of lipid-based systems have been encountered in topical and transdermal formulations applied on the skin’s surface throughout the literature. Among these systems, the first six categories are the most frequently encountered and chosen to be described in more detail [20]. A synopsis of the following examples, encompassing the characteristics of the nanostructures, the incorporated Active Pharmaceutical Ingredients (APIs), and the principal results, is presented in Table 1.

### 4.1. Liposomes

Liposomes are mono- or multi-lipid bilayer spherical structures with an aqueous core, consisting primarily of phospholipids and cholesterol. They are suitable for the incorporation of both hydrophilic and lipophilic drugs, either at their aqueous interior center or within the lipid bilayers. These types of nanocarriers exhibit a series of advantages, such as improved drug permeation with a prolonged-release profile and thus reduced side effects. Their site of action (topical or transdermal) can be determined via their particle size, finding numerous applications throughout the literature. Some examples of drugs incorporated are progesterone, topical anesthetics such as benzocaine, lidocaine, and tetracaine, betamethasone dipropionate, retinoids, etc. for the treatment of psoriasis [56]. Liposomes can also be utilized for the delivery of peptides such as cyclosporine A or for regenerative purposes to heal wounds.

Paclitaxel-loaded liposomes have been investigated as a potential therapeutic strategy for the treatment of squamous cell carcinoma. Findings from this study suggest that, compared to the administration of free paclitaxel, the liposomal formulation enhances drug penetration through the stratum corneum and increases antiproliferative activity, offering a promising approach for improving the management of squamous cell carcinoma [15]. In another study [57,58], a liposomal system incorporating DNA repair enzymes was evaluated for its efficacy in treating skin cancer. Specifically, the formulation contained T4 endonuclease V, a DNA repair enzyme derived from bacteriophage T4, which is capable of substituting the UV-damaged repair enzymes in human cells and initiating excision repair. In vitro experiments demonstrated that intracellular delivery of T4 endonuclease V successfully corrected DNA repair deficiencies. Clinical treatment of DNA repair-deficient skin conditions was performed using a T4N5 liposomal lotion, which showed enhanced removal of UV-induced DNA lesions within the first few hours post-application.

Hosny and Aldawsari developed avanafil, a phosphodiesterase-5 enzyme inhibitor used for the erectile dysfunction treatment, which was incorporated in a liposomal form in terms of enhancing its permeability. The results of the ex vivo permeation studies demonstrated that the developed liposomal distributions achieved a 4-fold increase in permeation compared to the conventional suspensions, while bioavailability was even up to 7 times higher [33]. Vitamin D3 and folic acid have also been investigated in liposomal formulations as UV-protective, antiaging, and regenerative agents, with promising results when compared with traditional ones [34,35].

Finally, pain management is considered one of the most critical medical issues; thus, the incorporation of non-anti-inflammatory agents in liposomes is still under investigation. Specifically, Franze et al. developed a lidocaine/CBD (cannabidiol) liposomal delivery system, where an increase in skin permeability was achieved allowing the release of the drug combination to the deeper skin layers. The aforementioned formulation is proved to be stable in long-term storage conditions for approximately one year, paving the way for new research in the field of pain control [36].

### 4.2. Solid Lipid Nanoparticles (SLNs)

**Figure 2:**
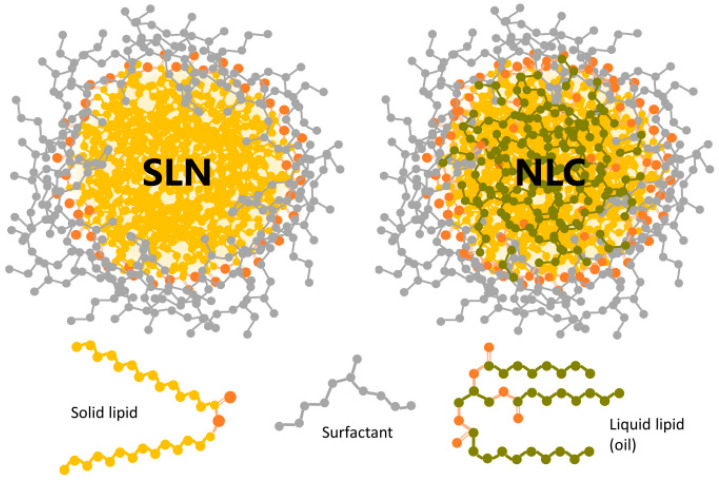
Schematic representation of an SLN and a NLC sterically stabilized with a neutral surfactant (gray) [59].

Solid lipid nanoparticles or SLNs are prepared by lipids that are solid at body temperature and consequently at room temperature stabilized by surfactants (Figure 2). Their size ranges from 50 to 2000 nm, exhibiting biodegradability, low toxicity, biocompatibility, and enhanced physical stability in comparison to the conventional liposomes. The active substance can be incorporated either in a dissolved or a dispersed state while SLNs are also considered promising carriers especially for lipophilic drugs (logP > 3) with molecular weights of above 500 Daltons. The system as a whole has the potential of forming an occlusive lipid film on the surface of the skin, prolonging the residence on the area and enhancing the release to the skin. This phenomenon is even more intense in anatomical areas where skin appendages are encountered [15].

Several key factors influence the drug loading capacity, release profile, and stability of lipid-based nanoparticles, including the polymorphic form of the lipid core and the length of the lipid chains. The presence of alkyl chains in the lipids exhibit a diverse range of packing arrangements such as α, β’, and β forms. These different packings ultimately influence the amount of loaded drug, stability profile, and drug release. For example, the presence of caprylic/capric triglyceride in the lipid core enhances physical stability by delaying recrystallization of the lipids. The degree of crystallinity can be assessed using X-ray diffraction (XRD), a widely employed technique for characterizing the crystalline structure of solid materials. Lipid composition also plays a critical role; highly crystalline lipids with well-ordered lattices tend to expel the drug, whereas more complex lipids—such as glycerol dibehenate, glyceryl monostearate, and glyceryl palmitostearate—composed of mono-, di-, and triglycerides, form imperfect lattices that allow for greater drug incorporation due to disrupted molecular packing [59,60].

Lipid nanoparticles are typically prepared by dissolving or dispersing the active compound within the lipid matrix, followed by the application of high energy to form nanostructures. In most of the techniques applied to large-scale batches, the drug is incorporated either in the melted lipid phase or in the solution of the lipid with organic solvents. In the first case, methods such as hot and cold high-shear homogenization and ultrasonication are included, while in the second one, solvent emulsification/evaporation, double emulsification, solvent diffusion, and solvent displacement find application [60]. Lately, the techniques of nanospray drying and hot melt extrusion are of great concern for scale-up and commercial continuous productions, as the nanoparticles are produced in a single step.

Regarding its in vivo efficacy, studies performed with cyclosporine A and calcipotriol-loaded lipid NPs demonstrated the lowest scores of eye inflammation and psoriatic symptoms in relation to the results collected from free medicines. Loteprednol etabonate (LE), a carbon-ester corticosteroid utilized for the treatment of inflammation, exhibited reduced adverse effects when incorporated in SLNs, while an increase in its action was achieved even at lower loading and dosage frequency [15]. Metformin, a known anti-diabetic agent, was loaded in solid lipid nanocarriers made of Tween 60, cholesterol, Span 60, and beeswax and evaluated in terms of efficacy and stability. This formulated carrier can increase the skin penetration with higher concentrations at the deeper layers of the skin, while regarding its stability; an optimization study is to be carried out to optimize the portions of the constituents [37]. Antifungal compounds such as amphotericin B [38], eugenol [39], fluconazole, griseofulvin [61], and miconazole nitrate [62] have been also incorporated in nanocarriers with promising results in terms of efficacy and adverse effects. SLNs also find many applications in the cosmeceutical field. Specifically, antioxidant molecules such as sesamol [40], silybin [41], and idebenone [63]; anti-wrinkle agents such as retinyl palmitate [64]; and many others have been developed in solid lipid nanocarriers with promising results. Benzoyl peroxide SLNs have significant advantages relative to commercial formulations, such as higher drug accumulation and reduced side effects and inflammation [15].

### 4.3. Nanostructured Lipid Carriers (NLCs)

Nanostructured lipid carriers (NLCs) are a form of nanocarriers that resemble the solid lipid ones mentioned earlier and have been developed to address the crystallinity issues and limitations that are related to the SLNs. For this reason, their core is formed from the combination of solid and liquid lipids, with some of the most common liquid lipids used for their preparation being oleic acid, sesame oil, almond oil, mixtures of medium-chain triglycerides, and many others. Compared to the remaining types of nanocarriers, they exhibit numerous benefits such as drug safety, controlled release of drugs, and enhanced bioavailability by increasing permeation either through the surface of the skin or its appendages. Specifically, NLCs interfere with the lipid bilayers causing lipid reorganization in the stratum corneum, thereby enhancing drug penetration. They also recover skin hydration by stopping transepidermal water loss and through film formation on the stratum corneum’s surface [42].

**Figure 3:**
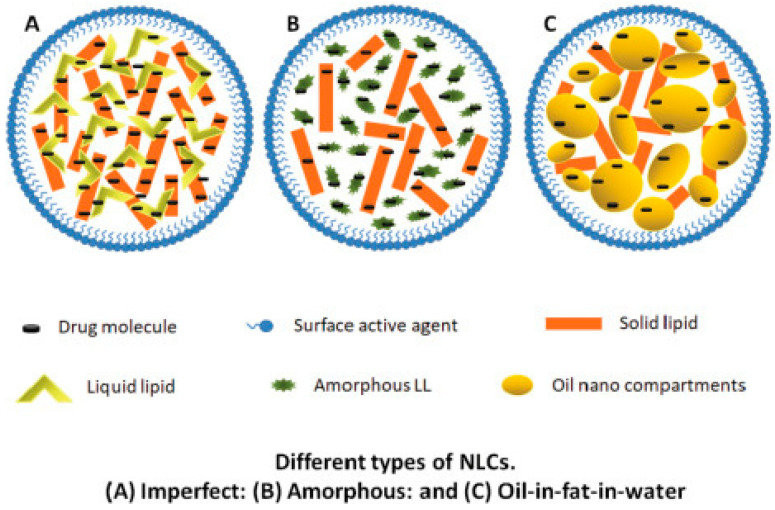
Morphological models of different types of nanostructured lipid carriers (NLCs) [42].

There are three main types of nanostructured lipid carriers: (I) Type 1 (imperfect) which acquires an imperfect crystal core structure due to the substitution of a portion of the solid lipids with liquid ones, and it is characterized by high drug loading and release properties, (II) Type 2 (amorphous) which contains medium-chain triglycerides as liquid lipids to maintain an α-polymorphic state after solidification, avoiding in this manner any crystallinity-related issues, and (III) Type 3 (multiple) in which small-sized oil droplets are dispersed in the solid lipid matrix resembling the structure of w/o/w emulsions and exhibiting enhanced drug loading and stability (Figure 3).

NLCs can be prepared by a variety of methods such as (a) high-pressure homogenization where the excipients are forced to pass through a micro-sized nozzle under high pressure, (b) high-shear homogenization and ultasonication where the contents of the lipid core are heated up to their melting point, mixed under high speeds with the aqueous phase to form an emulsion, and then ultrasonicated to reduce the particle size, (c) microemulsion technique where a microemulsion is initially prepared at high melting temperatures and then cooled down rapidly to form the NLCs, and (d) solvent emulsification/evaporation where lipids are dissolved in an organic solvent and then emulsified to the aqueous phase while evaporated to retrieve the precipitated particles [42].

NLCs find numerous applications in targeted skin delivery for many active substances. Several studies have investigated their usage in the treatment of skin conditions such as eczema, psoriasis, bacterial infections, and skin cancer. Specifically, studies have shown an enhanced efficiency of the lipophilic calcipotriol and hydrophilic methotrexate for psoriasis treatment, while N-acetyl glucosamine incorporated in Precirol^®^/Miglyol^®^ nanocarriers exhibited promising results in reducing the melanin distribution patterns caused by hyperpigmentation [42]. Acitretin incorporated in oleic acid and Precirol ATO 5 containing NLCs exhibited a significantly higher deposition of the active substance (*p* < 0.05) in the skin versus the conventional gel, while clinical findings showed significantly improved results in the treatment of psoriasis [43]. Topical NLC preparations have also been formulated for the incorporation of non-steroidal anti-inflammatory (NSAIDs) and anti-arthritic drugs. Flurbiprofen-loaded NLCs using coconut oil, soya lecithin, and stearic acid showed increased penetration through the skin and better therapeutic performance up to 24 h [44]. Other examples of incorporated NSAIDs are ketoprofen, meloxicam, nimesulide, and diclofenac sodium, for which enhanced anti-inflammatory action was demonstrated, accompanied by good skin tolerance and reduced toxicity. Several corticosteroids have also been investigated as potential candidates for NLC loading. The percentage of the active substance absorbed beyond the stratum corneum as well as the amount retained in the skin were enhanced by 0.1% difluctolone valerate-loaded nanoparticles in comparison to the commercial Nerisone^®^ cream [45]. Fluocinolone NLCs have demonstrated improved drug solubility and enhanced deposition within the epidermis—the primary site of keratinocyte hyperproliferation. Selective targeting of the epidermis might also eliminate any adverse effects associated with systemic exposure [65]. Several dermal applications of antifungals such as luliconazole, quercetin, and fluconazole can be encountered. Luliconazole, although highly effective against Trichophyton species, exhibits limited skin penetration and retention. With the aim of surpassing this obstacle, Baghel et al. [46] examined the development of such nanocarrier systems. In the case of fluconazole-loaded NLCs, a significant reduction in fungal load was observed, as evidenced by the lowest colony-forming unit (cfu/mL) count in treated subjects [42]. Studies on systemic targeting for the treatment of Alzheimer’s disease were also found throughout the literature. Donepezil- and rivastigmine-loaded nanocarriers in transdermal drug delivery systems (TDDSs) have been developed, showing better therapeutic efficacy both in in vitro studies and animal tests, paving the way for alternative options in dementia management [47,66]

NLCs are also of great interest in the field of cosmetic industry due to their advantages of improved skin hydration, occlusion, biocompatibility, skin targeting, and molecule stabilization. Retinol has found a wide use in cosmeceutical applications as an active compound against wrinkle formation. However, its molecule is susceptible to oxidation and therefore is not easily stabilized. On this basis, retinol-loaded NLCs were formulated and found stable after storage for four weeks at temperatures of 25, 40, and 50 °C. NLCs containing white willow bark extract, azelaic acid, and pathenol were also investigated in terms of enhancing skin hydration, with promising results [42].

### 4.4. Ethosomes and Transfersomes

**Figure 4:**
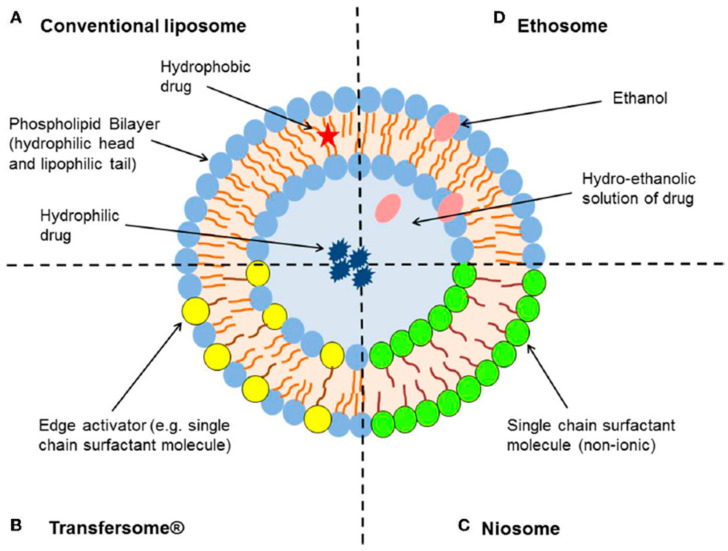
Schematic representation of the different types of lipid-based vesicular delivery systems [22].

Ethosomes are structurally similar to liposomes but are distinguished by their high ethanol content, typically ranging from 20 to 50% [20] (Figure 4).

**Figure 5:**
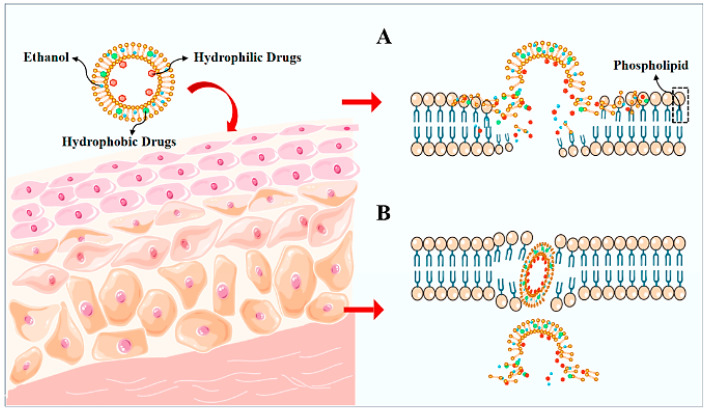
A schematic representation of the main permeation mechanisms of ethosomes: (**A**) the ethanol effect and (**B**) the ethosome effect [67].

This ethanol component imparts deformability to the vesicles, enhancing their ability to penetrate the skin. Ethanol is a well-established permeation enhancer and is believed to work synergistically with both the vesicular structure and the lipids within the skin. Specifically, ethosomes initially penetrate the skin by disrupting the highly organized layer of stratum corneum via their contained ethanol (“the ethanol effect”). The interaction of ethanol with the polar heads of the lipid bilayers increases their fluidity while decreasing their density, thereby resulting in nanocarriers with flexible features and the ability to penetrate into deeper tissues. Lastly, the fusion taking place between the phospholipids present in the SC and the ethosomal vesicle leads to enhanced drug delivery (“the ethosome effect”) [48,67] (Figure 5).

A number of ethosome-based formulations can be applied for the treatment of skin pathologies such as psoriasis, dermatitis, acne vulgaris, and skin cancer or for the systemic treatment of inflammation, pulmonary diseases, bacterial, viral, or fungal infections, and many others. In more detail, psoralen, a photosensitive coumarin used for the treatment of the skin symptoms of psoriasis, was loaded in ethosomes to assess its efficacy. The study demonstrated a 6.56-fold increase in skin deposition of the active compound, along with enhanced permeation, compared to a conventional formulation [48]. Topical photodynamic therapy (PDT) with 5-aminolevulinic acid (ALA) is a treatment option to deal with the skin symptoms of psoriasis. The main mechanism of PDT is the modulation of cellular functions without causing cellular death. ALA specifically acts as a prodrug of the photosensitizer protoporphyrin IX (PpIX), formed after the in vivo application of ALA. Studies in murine models with hyperproliferative skin have shown that ALA-loaded nanocarriers improve ALA penetration and subsequently increase PpIX formation, while also reducing local TNF-α levels when compared to a standard aqueous ALA solution [68]. Scientists have also incorporated methotrexate, an anti-psoriatic and anti-neoplastic agent, in ethosomes to assess if improved efficacy can be achieved. The results of the study revealed enhanced efficacy with lower toxicity accompanied by a better stability profile [49]. In terms of treating bacterial, viral, or fungal infections, active substances such as erythromycin [50], acyclovir [51], and fluconazole have been loaded in ethosome carriers and have been examined with regard to their penetration to the systemic circulation [69]. In all cases, enhanced skin penetration was detected when compared with conventional formulations. Pulmonary-targeted active substances have also been studied to be enclosed in ethosomes to enhance their systemic absorption from the skin. Specifically, ligustrazine-loaded ethosomes have shown promising results when administered through the transdermal route, with the ethosome patch group achieving a 2.09-fold increase in comparison to the oral route and a 2.12-fold increase in comparison to that of the conventional patches. The relative bioavailability was about 209.45%, while the oral one was only up to 98.63% [52].

Transfersomes, known for their high flexibility, are elastic vesicles composed of the standard bilayer and an edge activator (e.g., single-chain surfactant molecule), which is responsible for the weakening of the lipid bilayer and the increased deformability (Figure 4). When applied on the surface of the skin, the transfersomes deform to pass through the keratinized layer to finally reach the deeper layers of the skin and the systemic circulation [20].

Transfersomes have been examined as vehicles for a variety of small molecules such as steroids, NSAIDs, and topical anesthetics, as well as peptides and proteins. Transfenac^®^, a topical formulation containing diclofenac, has been shown to achieve therapeutically promising drug concentrations in target tissues. In preclinical studies, administration of a transfersome dose ranging from 0.25 to 2.00 mg/kg of rat body weight resulted in muscle tissue concentrations of 0.5 to 2.0 µg/g. In contrast, a conventional hydrogel formulation yielded concentrations below 0.5 µg/g [70]. Triamcinolone acetonide, a corticosteroid, was evaluated in terms of distribution and bioavailability in comparison to the commercially available lotion and cream products. The suppression of edema in mice was used as an indicator of the efficacy of the formulations. The results of the study indicated that the same biologic effect could be achieved with a 25-fold lower loaded dose of the active substance in the transfersomes. In this manner, a reduction in side effects is possible, without compromising the efficacy of the product [53]. Similar results were also recorded for dexamethasone-loaded transfersomes, where no lag time was detected after the application of the product, indicating the rapid release of the active substance to the site of action [54]. It is also reported that transfersomes can deliver similar amounts of insulin to the ones administered with subcutaneous injection. Insulin-loaded transfersomes composed of phosphatidylcholine were assessed after their applications in both mice and humans, and comparable levels to the ones of subcutaneous injections were measured, with only a difference in the lag time [55]. The potential of transfersomes for non-invasive vaccine delivery has also been investigated, with similar outcomes in the generation of antibodies in comparison to the standard used vaccines.

**Figure 6:**
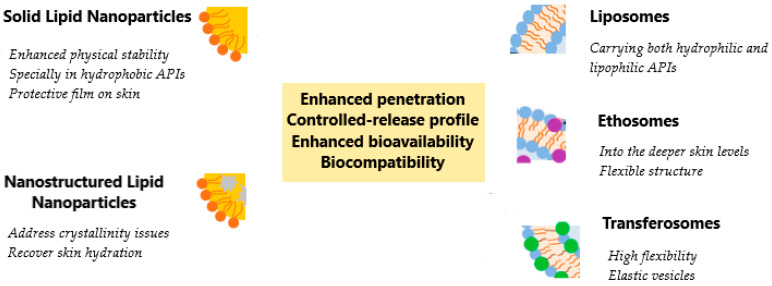
A schematic representation of the advantages of the lipid-based drug delivery transdermal systems.

A summary of the advantages of each of the lipid-based drug delivery transdermal systems categories is illustrated in Figure 6, while some of their main common advantages are enhanced penetration and bioavailability, a controlled release profile, and biocompatibility.

## 5. Fast Clinical Translation of Lipid-Based Formulations for Transdermal Administration

Lipid-based formulations have already been marketed for the last thirty years as anticancer or antifungal medicines. The technology transfer from lab-scale to industrial batches, as well as the microfluidic-based techniques, is responsible for the fast clinical translation of liposomes and other lipid-based formulations. The preclinical models, i.e., human skin explants or reconstructed human epidermis, accelerate the evaluation of the prepared lipid-based formulations for transdermal administration [6].

Several preclinical studies have been conducted in the last decade for the enhanced transdermal delivery of diclofenac encapsulated in several lipid-based nanoformulations for the treatment of pain and inflammation [71,72].

A clinical study of 66 postmenopausal Brazilian women with climacteric symptoms of natural menopause who received progesterone and estradiol formulated in NLC in the forearm daily for 60 months to mimic the normal ovarian secretory pattern reveals the usage of the fast clinical translation of the aforementioned systems [73]. Lidocaine nanosystem behavior is also comparable to the marketed products for dermal delivery [74].

Last but not least, the combination of microneedles with liposomes has already been reported in the recent literature, where a glucose-responsive insulin delivery microneedle (MN) array patch is loaded with liposome nanoparticles containing glucose transporters bound with glucosamine-modified insulin. The results were very optimistic for the control of the levels of glucose and insulin in blood [75]. The clinical status of several lipid-based drug delivery systems for transdermal administration is presented in Table 2.

## 6. Quality by Design Approach for the Design and the Development of Lipid-Based Drug Delivery Systems

**Figure 7:**
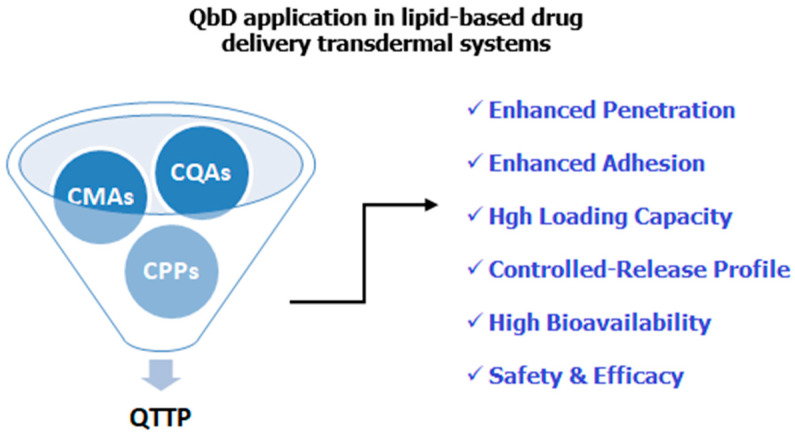
The QbD approach towards the design and development of the lipid-based drug delivery transdermal systems.

Researchers in academia, regulatory bodies, and pharmaceutical industries are working towards the quality assurance and the continuous improvement in the pharmaceutical products. Towards these efforts, evolving the Quality by Design (QbD) within the product development for the rational design of the formulation and manufacturing process embraces this scope. Based on the ICH Guidelines, i.e., Q8 for pharmaceutical development, Q9 for quality risk management, and Q10 for pharmaceutical quality systems, the QbD can define the Quality Target Product Profile (QTPP), the critical quality attributes (CQAs), the critical material attributes (CMAs), and the Critical Process Parameters (CPPs) of the product and further analyze the risk factors [76]. The QbD approach can be also applied towards the rational design and the development of lipid-based drug delivery systems, as it is presented in Figure 7.

The critical quality attributes (CQAs) of the lipid-based nanoparticles that influence their performance during skin administration include particle size, particle size distribution, particle shape, and surface chemistry, taking also into account some critical material attributes (CMAs). The most critical aspect of a successful transdermal delivery of a lipid-based nanosystem is the effective skin permeation; thus, the choice and the optimization of the CQAs and CMAs during the formulation process should target this scope.

In terms of parameters such as size and size distribution, in general, nanoparticles larger than 600 nm in size are unable to penetrate to deeper skin layers, remaining only on or in the stratum corneum. Nanoparticles of size of 300 nm or less can penetrate and thus deliver more deeply, via the transfollicular route [77], in a size-dependent manner (the smaller the size, the higher the follicular transport, with more preferable embodiments at 10 to 210 nm sizes) [78]. Nanoparticles of 100 nm or less are suitable for deep skin penetration and allow the onset of action [79]. In particular, vesicles smaller than 70 nm can be found in both the viable epidermal and dermal layers via lipid transepidermal pathways, while those smaller than 36 nm can be absorbed via aqueous pores [22]. The combination of smaller sizes and higher vesicular elasticity has been proven to lead to a significant permeation enhancement effect. Two representative examples are the transferosomes (with a smaller vesicular size, typically <300 nm, and higher elasticity, typically 5–8 times higher compared to conventional liposomes) and the ethosomes (with a smaller vesicular size, typically <300 nm, and higher elasticity, typically 10–30 times higher compared to conventional liposomes), being ideal nanovesicles for skin permeation applications [28,80].

The mode of the interaction between the nanoparticles and the lipid bilayer is strictly influenced by the particle’s shape, volume, contact surface, local curvature at the point of contact, and initial orientation, therefore affecting the nanoparticle ability to permeate the lipid bilayer. For example, an ellipsoid particle is able to penetrate a lipid bilayer up to five times faster [81].

Surface charge is another key factor affecting the drug permeation and the in vivo behavior of nanoparticles after transdermal administration. Taking into account the physiology of the skin tissue, the normal skin has a net negative charge, in combination with the presence of sebum. Several recent studies suggest that lipid nanoparticles and sebum lipids should be attracted to each other to maximize drug penetration; therefore, positively charged nanoparticles are favored to exhibit better targeting [82]. Abdel-Mottaleb et al. [78] concluded that the presence of charge could enhance skin adhesion and the interaction of nanosystems, leading to a higher therapeutic effect on inflamed skin. In another studies, cationic nanoparticles are attracted by the negatively charged skin membrane [83], to transfer plasmid DNA to the skin [84], as well as cationic nanovaccines of transferosomes into microneedles escape endocytic compartments, allowing antigen processing via an MHC-I presentation pathway and increasing lymph node accumulation [85]. For the pH-responsive liposomes, the pH triggers several morphological changes, which lead to API release. The skin pH is acidic, around 5.5, while in deeper layers, the pH is higher. Taking into account these physiological conditions, the pH-responsive lipid-based nanoformulations are of particular interest for transdermal controlled drug release [86]. As mentioned above, the zeta potential is a crucial physicochemical parameter for the colloidal stability of lipid-based nanoformulations. The interaction with skin components and the penetration efficiency are also affected by the zeta potential. For instance, the positive charge of lipid-based nanoparticles improves the adhesion to the skin, the penetration, and the permeation because stronger electrostatic interactions are developed with the negatively charged components of the stratum corneum [77]. However, some negatively charged skin components have also been referred to. For example, compared with classic liposomes and ethanolic drug solutions, ethosomes are considered to increase the transdermal permeability of the encapsulated drug, due to their negative charge, in a higher ethanol concentration, leading to a continuous high transdermal flux [87].

Molecular dynamics simulations suggest that semi-hydrophilic particles can be adsorbed on the surface of the membrane bilayer, while hydrophobic nanoparticles penetrate the lipophilic core of cell membranes [82]. Yang et al. [88] developed a nanoemulsion for the delivery of retinyl that, due to its hydrophobic nature, exhibited highly enhanced results in the epidermis and dermis compared to conventional emulsions.

Moreover, the type of materials plays a key role in the later interaction of the nanoparticle with the skin. Starting from the typically used lipid, phosphatidylcholine, a well-established material of lipid nanocarriers, can act as a permeation enhancer for skin delivery of the drugs, being controlled by their phase transition temperature. In contrast, while the cholesterol increases membrane rigidity, it can downgrade the permeation through the skin [89]. Various fatty acids, being used in the formulation of SLNs and NLCs, can act as excellent skin penetration enhancers due to their ability to interact with stratum corneum lipids. Their penetration degree is controlled by their chemical structure, the length of the alkyl chain, and the degree of saturation [82]. The matrices of solid lipids are able to promote an extended and controlled drug release due to the limited drug diffusion from the matrix, being suitable for transdermal administration due to the small size and thus easier penetration. Apart from the lipids, the surfactants are considered to be permeation skin enhancers in a mode controlled by their hydrophilic–lipophilic balance (HLB), with the niosomes being representative examples. The HLB also affects the stability of the formulation and the complexation/encapsulation/incorporation of the API into the lipid-based nanocarrier [30].

Numerous other additional CQAs have been nominated for lipid-based nanosystems and are also applied in transdermal cases. Some of the typical CQAs are encapsulation efficiency, loading capacity, internal volume, phase transition temperature, morphology/lamellarity, etc. Lipids assays and degradation products of lipid component identification can affect particle size, polydispersity index, EE, loading capacity, and zeta potential, and hence may influence the therapeutic efficacy and the safety profile of the drug product [90].

Adhesion properties and elasticity are key parameters for skin permeation and therefore should be investigated during product and process development of transdermal products [89,91]. Elasticity allows the nanoparticle entrance via the small skin pores [71]. Spreadability is a quality attribute specific to topical formulations, such as ethosomes, depending on the viscosity of the formulation and has an impact on topical drug availability, and hence in the product efficacy. In transdermal delivery, viscosity may influence the drug diffusion rate at the microstructural level and, consequently, the delivered final dose and, therefore, the bioavailability [92].

**Figure 8:**
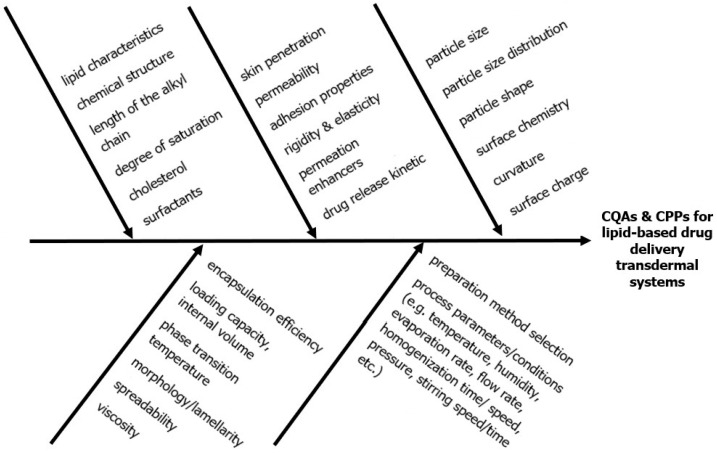
Ishikawa diagram presenting the CQAs and CPPs for lipid-based drug delivery transdermal systems.

Finally, in the case of nanoparticles, being enclosed into transdermal matrices, like reservoir-gradient, matrix diffusion-controlled or membrane-moderated systems, the CQAs of these matrices themselves should also be taken into account and typically include the following: peel adhesion, tack, release liner peel strength, shear strength, cold flow, residual solvents, residual monomers, microbial limits, the uniformity of dosage units, assay, permeation enhancer content, impurities and degradants, in vitro drug release profile, and (if present) preservative/antioxidant content [93]. In the Ishikawa diagram of Figure 8, the main CQAs along with CMAs are presented.

## 7. Preparation and Characterization Methods of Lipid-Based Drug Delivery Systems

A precise definition of all the CPPs that are being evolved during the manufacturing process should take place during the development of the lipid-based drug delivery systems that would also help towards the selection of the most proper manufacturing method (Figure 6). Moreover, the manufacturing environment is more challenging than that at smaller scales, as there is great difficulty in maintaining stable conditions of temperature, humidity, aseptic operation, etc., and achieving batch repeatability within the specifications.

Microfluidization techniques, the bubble method, the ether ejection method, and reverse phase evaporation are some of the main industrial techniques referred to as nanosystems intended for transdermal delivery [94]. Depending on the method, different CPPs can be identified and should be investigated during the process developments. Some of them are considered to be evaporation rate, flow rate, homogenization time/speed, humidity, number of cycles, phase addition order, pressure, stirring speed/time, temperature, etc. [91].

Apart from the advantages described in the previous paragraphs, the rational development of effective transdermal lipid-based products remains a challenge for the pharmaceutical industry. As it is analyzed above, the multifactorial formulation that should be taken into account in order to achieve effective skin permeation, along with the limitations of the already existing manufacturing methods, has restricted the market authorization of many products. Some of the manufacturing limitations include the limited scalability, the high batch-to-batch variance, and the increased stability requirements. The elevated development costs make it challenging to maintain high standards for quality, while it should be assured that appropriate environmental parameters (light, temperature, and humidity) are met during production, packaging, storage, and ultimate use [95].

The thorough characterization of lipid-based nanoformulations for transdermal administration is of paramount importance for their rational development as well. The interactions between lipid components and the API can be evaluated by thermal analysis techniques. Firstly, the physicochemical characteristics, i.e., size distribution, zeta potential, morphology, and shape, are crucial for uniformity and skin penetration ability, as well as stability. The drug loading, release, and stability studies are useful for the efficiency of API encapsulation and short- and long-term stability and storage conditions, respectively. The pH and the viscosity also reflect the formulation stability and applications in skin layers. After the detailed physicochemical characterization, the skin permeation and retention tests follow. For in vitro permeation tests, human or animal skin in Franz cells is widely used. Skin homogenization and API quantification for ex vivo skin retention are applied.

## 8. Regulatory Aspects of Lipid-Based Drug Delivery Systems

### 8.1. Characterization Requirements by Regulatory Framework

In the regulatory sense, some of the main requirements, among others, for the liposomal formulations, including the topically applied ones, are summarized below: chemical quality of lipids and other non-lipid components, lipid content, total drug substance content, encapsulated/free fractions of a drug substance, distribution of a drug substance within a liposome (e.g., surface, bilayer, interior), lipid-phase transition temperature, drug leakage from a carrier, degradation products of lipids and drug substances, stability of drugs, lipids, and other critical excipients in the finished product, particle size distribution and net charge (zeta potential), drug product viscosity, in vitro drug release, liposome integrity changes in response to environment or addition of other excipients, and stability of liposomal systems during storage and in-use conditions. As long as the manufacturing process is concerned, the identification of its key steps and its suitable controls are required, for example, monitoring the drug/lipidic moiety ratio at relevant manufacturing steps, description of the process, mechanism of liposomal drug loading, etc. [96].

### 8.2. Challenges and Limitations of Regulatory Framework

Despite the plurality of the clinical trials registered in both the USA and the EU, few pharmaceutical products containing lipid vesicles (e.g., PevarylR Lipogel, MaxileneR cream, LipoxysanR, Supra-virR cream) have been market-authorized as topically applied pharmaceutical products [96]. Apart from the manufacturing aspects, the regulatory framework also creates challenges to their market authorization. For example, when major post-marketing variations are required, the complexity of a nanoparticulate formulation, even more via the skin route of administration, limits the ability of bioequivalence studies to assess the clinical equivalence of two products, requiring more expensive clinical trials [96]. Furthermore, the FDA requires an analytical study to be performed on the hair follicle penetration of topical nanoformulated products and on how the permeation mode is affected by the skin conditions (e.g., intact, damaged, diseased), while the EMA has suggested that permeation kinetics studies, like the in vitro permeation studies, for topical nanomedicine products be performed not only during the pharmaceutical development, but also as supportive studies for comparison reasons with the conventional products [96]. However, due to the variations in animal and human skin tissue, the current methods for bioequivalence access of transdermal delivery systems are not fully standardized, while the randomized controlled clinical trials are expensive and time-consuming and require a large number of participants that comply with ethical considerations [95]. A number of guidelines, guidance for industry, and reflection papers have been published by both the FDA and the EMA on the rational development of these products, covering issues on the presence of nanomaterial ingredients [97,98,99,100], as well as about product development and quality considerations of transdermal and topical delivery systems [101,102] that should be taken into account.

## 9. Conclusions

The transdermal route could be a beneficial route of administration, especially due to its non-invasive character, although there are still obstacles impeding its usage. Lipid-based drug delivery systems could be promising candidates for pharmaceutical applications enabling efficient transcutaneous delivery due to their biocompatibility, versatility, and effective skin penetration. The most recent developments of lipid-based nanoparticles via the transdermal route of administration are presented. The physicochemical characteristics of all lipid-based nanoparticles are considered as CQAs and play a key role in their effectiveness. Studies regarding the added value and the applications of liposomes, ethosomes, solid lipid nanoparticles, transferosomes, and nanostructured lipid carriers showed how these systems enhance effectiveness and reduce adverse drug reactions. The thorough characterization of lipid-based nanoformulations for transdermal administration is of paramount importance for their rational development and fast clinical translation. Considering patients’ unmet needs, the critical quality attributes, the existing challenges, and the regulatory framework are roadmaps for formulation scientists in order to design and develop transdermal lipid-based nanomedicines.

## Figures and Tables

**Table 1:** A synopsis of the main lipid-based drug delivery systems for transdermal administration and their properties.

Vesicular Type	Physicochemical Characteristics	API	Application	Key Findings	Reference
Liposomes	**Particle size**: 600–900 nm (MLVs) and 300–600 nm (ULVs) **EE%**: 40–95% (MLVs) and 20–60% (ULVs)	Avanafil	Erectile dysfunction	Four-fold increase in permeation and seven-fold in bioavailability compared to the conventional suspensions	[33]
	**Particle size**: 100–150 nm **EE%**: 85% **PDI**: 0.1–0.3	Vitamin D3	UV-protective, antiaging, and regenerative agents	Increased retention of Vitamin D3 in skin; improved skin appearance	[34]
	**Particle size**: 120 **EE%**: 10% **PDI**: 0.3 **Zeta potential**: −27 mV	Folic acid	UV-protective, antiaging, and regenerative agents	Liposome stabilization at room temperature; transdermal penetration enhancement	[35]
	**Particle size**: 80–100 nm **EE%**: 60–95% (CBD) and 50–90% (LD)	Lidocaine (LD)/cannabidiol (CBD)	Pain relief	Improved skin penetration; 1-year storage stability	[36]
Solid Lipid Nanoparticles (SLNs)	**Particle size**: 60–100 nm **EE%**: 40–80% (cyclosporine A) and 80–100% (calcipotriol) **PDI**: 0.2	Cyclosporine A and calcipotriol	Psoriasis and inflammation treatment	Low scores of eye inflammation and psoriatic symptoms	[15]
	**Particle size**: 204 nm **EE%**: 33% **PDI**: 0.6 **Zeta potential**: −5 mV	Metformin	Topical anti-inflammatory	Enhanced penetration to deeper skin layers	[37]
	**Particle size**: 111–416 nm **EE%**: 73–94% **PDI**: 0.1–0.3 **Zeta potential**: −5 to −23 mV	Amphotericin B	Antifungal therapy	Enhanced efficacy and reduced adverse events	[38]
	**Particle size**: 363–423 nm **PDI**: 0.4–0.5	Eugenol	Antifungal therapy	Enhanced efficacy and reduced adverse events	[39]
	**Particle size**: 179–279 nm **EE%**: 54–76% **PDI**: 0.2–0.3 **Zeta potential**: −23 to −34 mV	Fluconazole	Antifungal therapy	Enhanced efficacy and reduced adverse events	
	**Particle size**: 128 nm **EE%**: 88% **PDI**: 0.3	Sesamol	Antioxidant activity for thetreatment of skin cancer	Enhanced bioavailability up to the desired anticancer effect	[40]
	**Particle size**: 139 nm **EE%**: 91% **PDI**: 0.3 **Zeta potential**: −30 mV	Silybin	Antioxidant activity against irritant contact dermatitis	Enhanced skin delivery and therapeutic efficacy in treating irritant contact dermatitis compared to conventional formulations	[41]
Nanostructured Lipid Carriers (NLCs)	**Particle size**: 260–320 nm **PDI**: 0.3 **Zeta potential**: −42 to −45 mV	Calcipotriol and methotrexate	Psoriasis treatment	Enhanced skin permeation and negligible irritation	[42]
	**Particle size**: 150–300 nm **EE%**: 55–65% **PDI**: <0.2 **Zeta potential**: −30 to −35 mV	Acitretin	Psoriasis treatment	Improved therapeutic response (reduced erythema) with mild adverse effects versus commercial gel	[43]
	**Particle size**: 214 nm **EE%**: 93% **PDI**: 0.2 **Zeta potential**: −31 mV	Flurbiprofen	Treatment of osteoarthritis, rheumatoidarthritis	Reduction in erythema along with maintenance of the anti-inflammatory effect versus the commercial gel	[44]
	**Particle size**: 218 nm **EE%**: 65% **PDI**: 0.3	Difluctolone valerate	Treatment of skin diseases such as psoriasis, acne, tinea, ulcers, and discoid lupus erythematosus	High targeting of the API in the stratum corneum (SC) and limited systemic side effects versus Nerisone^®^ cream	[45]
	**Particle size**: 173 nm **EE%**: 90% **PDI**: 0.3 **Zeta potential**: −57 mV	Luliconazole	Antifungal therapy	Enhanced antifungal activity with no irritation compared to the commercial formulation	[46]
	**Particle size**: 170–240 nm **EE%**: 99% **PDI**: 0.3 **Zeta potential**: −40 to −55 mV	Donepezil	Alzheimer disease	Higher skin permeation with limited adverse effects	[47]
Ethosomes	**Particle size**: 120–150 nm **EE%**: 85–95%	Psoralen	Psoriasis treatment	6.56× higher deposition vs. control	[48]
	**Particle size**: 143 nm **EE%**: 69% **PDI**: 0.1	Methotrexate	Psoriasis and cancer therapy	Improved efficacy, lower toxicity	[49]
	**Particle size**: 123 nm **EE%**: 79%	Erythromycin	Anti-bacterial treatment	Significantly improved anti-bacterial action vs. free drug in in vitro tests; non-toxic to dermal cultured fibroblasts	[50]
	**Particle size**: 120 nm **EE%**: 80–90% **Zeta potential**: +20 mV	Acyclovir	Anti-viral treatment	Enhanced permeability of ethosome formulations	[51]
	**Particle size**: 79 nm **EE%**: 86% **PDI**: 0.1	Ligustrazine	Pulmonary treatment	AUC 2.09-fold increase vs. oral; 209% relative bioavailability	[52]
Transfersomes	**Particle size**: 100–200 nm	Triamcinolone acetonide	Corticosteroid	25× lower dose for same efficacy	[53]
	**Particle size**: 90–200 nm **EE%**: 60–90%	Dexamethasone	Corticosteroid	Improved transdermal delivery, prolonged release with no lag time	[54]
	*No data available due to patent restrictions*	Insulin	Diabetes	Comparable levels to subcutaneous injections	[55]

**Table 2:** The clinical status of lipid-based drug delivery systems for transdermal administration.

Lipid-Based Formulation	API	Indication	Added Value	Clinical Status	Reference
Liposomes + microneedles	Insulin	Diabetes		Preclinical studies	[75]
SLNs	Lidocaine	Local anesthesia	Longer local anesthesia	Preclinical studies	[74]
Transfersomes	Diclofenac	Anti-inflammatory	Increased tissue concentration and permeation	Clinical study	[71,72]
NLCs	Estradiol	Hormone therapy	92.5% symptom relief, hormone normalization	Preclinical studies	[73]

## Data Availability

No new data were created or analyzed in this study. Data sharing is not applicable to this article.
